# Soluble Adhesion Molecules in Patients Coinfected with HIV and HCV: A Predictor of Outcome

**DOI:** 10.1371/journal.pone.0148537

**Published:** 2016-02-05

**Authors:** Teresa Aldámiz-Echevarría, Juan Berenguer, Pilar Miralles, María A. Jiménez-Sousa, Ana Carrero, Daniel Pineda-Tenor, Cristina Díez, Francisco Tejerina, Leire Pérez-Latorre, José M. Bellón, Salvador Resino

**Affiliations:** 1 Infectious Diseases and HIV Unit, Hospital General Universitario Gregorio Marañón, Madrid, Spain; 2 Instituto de Investigación Sanitaria Gregorio Marañón (IiSGM), Madrid, Spain; 3 National Centre of Microbiology, Instituto de Salud Carlos III, Majadahonda, Madrid, Spain; Chiba University, Graduate School of Medicine, JAPAN

## Abstract

**Background:**

Higher serum levels of adhesion molecules (sICAM-1 and sVCAM-1) are associated with advanced liver fibrosis in patients coinfected with human immunodeficiency virus and hepatitis C virus. We assessed the relationship between serum levels of adhesion molecules and liver-related events (LRE) or death, in coinfected patients.

**Methods:**

We studied clinical characteristics and outcomes of 182 coinfected patients with a baseline liver biopsy (58 with advanced fibrosis) and simultaneous plasma samples who were followed for median of 9 years. We used receiver-operating characteristic (ROC) curves to calculate optimized cutoff values (OCV) of sICAM-1 and sVCAM-1, defined as the values with the highest combination of sensitivity and specificity for LRE. We used multivariate regression analysis to test the association between OCVs of sICAM-1 and sVCAM-1 and outcomes. The variables for adjustment were age, HIV transmission category, liver fibrosis, baseline CD4+ T-cell counts, antiretroviral therapy, and sustained virologic response (SVR).

**Results:**

During the study period 51 patients had SVR, 19 had LRE, and 16 died. The OCVs for LRE were 5.68 Log pg/mL for sICAM-1 and 6.25 Log pg/mL for sVCAM-1, respectively. The adjusted subhazard ratio (aSHR) (95% confidence interval [CI]) of death or LRE, whichever occurred first, for sICAM-1 and sVCAM-1 > OCV were 3.98 ([1.14; 13.89], *P* = 0.030) and 2.81 ([1.10; 7.19], respectively (*P* = 0.030).

**Conclusions:**

Serum levels of sICAM-1 and sVCAM-1 can serve as markers of outcome in HIV/HCV-coinfected patients. Therapies targeting necroinflammatory damage and fibrogenesis may have a role in the management chronic hepatitis C.

## Introduction

Progression of chronic hepatitis C infection to cirrhosis and end-stage liver disease is more frequent and rapid among individuals infected by human immunodeficiency virus (HIV) than in those not infected by HIV [1]. The determinants of this faster progression of hepatitis C in patients coinfected by HIV and hepatitis C virus (HCV) are not fully understood; however, immunosuppression, alcohol consumption, and, probably, direct action of HIV have been identified as major contributing factors [2].

Immune dysfunction and systemic inflammation are hallmarks of the natural history of HIV infection [3], and both may be exacerbated in HIV/HCV-coinfected patients [4]. Chronic immune activation accompanied by an inflammatory response and cytokine production has been described in chronic HCV infection [5, 6]. Immune dysfunction and inflammation are also characteristic of end-stage liver disease, regardless of etiology [7].

Intercellular adhesion molecule 1 (ICAM-1) and vascular cell adhesion molecule 1 (VCAM-1) are transmembrane proteins belonging to the immunoglobulin superfamily that are induced on the endothelium and/or cells of the immune system by various cytokines at sites of inflammation. They are responsible for adhesion and/or migration of leukocytes and thus control the passage of these cells through tissues [8].

Adhesion to and penetration of sinusoidal vascular endothelium are critical for leukocyte migration and accumulation at sites of liver inflammation. Passage of leukocytes is controlled by interactions between adhesion molecules on leukocytes and corresponding ligands on endothelial cells [9]. ICAM-1 and VCAM-1 play a major functional role through their expression on cell surfaces, although a small number are cleaved from sites of expression and may be found in soluble forms (sICAM-1 and sVCAM-1) in serum [10]. These soluble cellular adhesion molecules are released from the cell surface after activation of cytokines and induction of inflammatory response [11]. In HCV-monoinfected patients, higher serum levels of sICAM-1 and sVCAM-1 are associated with higher grades of necroinflammatory activity and more advanced stages of liver fibrosis [12, 13]. We previously showed higher levels of sICAM-1 and sVCAM-1 in HIV/HCV-coinfected patients than in healthy HIV-negative controls and found a trend towards higher levels of these molecules in patients with more advanced stages of liver fibrosis [14]. Our aim was to assess the relationship between serum levels of sICAM-1 and sVCAM-1 and clinical outcomes, including liver-related events and death, in HIV/HCV-coinfected patients.

## Materials and Methods

### Design and Patient Selection

Baseline clinical and laboratory parameters from all HIV/HCV-coinfected patients who underwent liver biopsy in our institution after 2000 were prospectively recorded in an *ad hoc* database. For this study we retrospectively analyzed the follow-up of 182 HIV/HCV-coinfected patients from the HIV outpatient clinic of our institution that were previously reported in a cross-sectional study that examined the association of sICAM-1 and sVCAM-1 serum levels with liver fibrosis stage at the time of liver biopsy [14]. The patients were potential candidates for interferon and ribavirin who had both a liver biopsy specimen and simultaneous frozen plasma samples taken between 2000 and 2007. All patients, had a positive qualitative HCV RNA assay, negative hepatitis B surface antigen, CD4+ lymphocyte count higher than 200 cells/uL, stable combination antiretroviral therapy (cART) or no need for cART; and absence of active opportunistic infections, active drug addiction, other concomitant diseases or conditions such as diabetes, nephropathies, autoimmune diseases, neoplasia, and contraindications for interferon alpha and ribavirin therapy. All patients gave their written informed consent to participate in this study, and the Institutional Ethics Committee of Hospital General Universitario Gregorio Marañón approved the study.

### Clinical and Laboratory Data

Baseline data included age, gender, alcohol consumption, HIV transmission category, Centers for Disease Control and Prevention clinical category, nadir CD4+ T-cell count, and cART. We considered intake of >50 grams of alcohol per day for ≥12 months as high. A blood sample was taken from each patient before biopsy to analyze complete blood counts, coagulation tests, liver panel, basic metabolic panel, CD4+ T cells, plasma HIV-RNA, and plasma HCV-RNA. In addition, a serum sample was immediately frozen and stored at –70°C for further assays.

Outpatient percutaneous liver biopsies were performed by PM and JB following widely accepted recommendations [15]. Liver biopsy samples were examined by two pathologists who agreed on the scoring of fibrosis following the criteria of the METAVIR Cooperative Study Group [16], as follows: F0, no fibrosis; F1, portal fibrosis; F2, periportal fibrosis or rare portal-portal septa; F3, fibrous septa with architectural distortion and no obvious cirrhosis (bridging fibrosis); and F4, definite cirrhosis.

The study ran from the day of the liver biopsy (baseline) until death or the last follow-up visit. The administrative censoring date was June 30, 2013. Follow-up information included treatment of HCV infection and response, liver-related events, and mortality.

Sustained virologic response (SVR) was defined as an undetectable serum HCV-RNA level 24 weeks after discontinuation of interferon and ribavirin. The liver-related events analyzed included ascites, hepatic encephalopathy, variceal bleeding, and hepatocellular carcinoma. Ascites was confirmed by paracentesis and/or ultrasound. Hepatic encephalopathy was established on clinical grounds after the reasonable exclusion of HIV-associated encephalopathy based on clinical findings, laboratory parameters, and neuroimaging techniques. The source of gastroesophageal bleeding was confirmed by endoscopy whenever possible. Diagnosis of hepatocellular carcinoma was based on noninvasive imaging tests or pathology findings [17]. All the information related to death was reviewed by TA and JB, who classified death as follows: i) liver-related death, when the train of events that ended in death was caused by liver decompensation or hepatocellular carcinoma; ii) AIDS-related death, when death was directly related to an AIDS-defining condition; and iii) non–liver-related non–AIDS-related death.

### Serum Markers Analyzed

Serum levels of sICAM-1 and sVCAM-1 were measured using Multiplex kits (LINCOplex^TM^; LINCO Research, St. Charles, Missouri, USA) and the Luminex 100™ analyzer (Luminex Corporation, Austin, Texas, United States). These markers were detected in 100% of the samples tested.

### Statistics

The primary endpoint was the occurrence of liver-related events. For patients who had more than one liver-related event, only the first was included in the analysis. We also assessed death and the composite variable of death or liver-related events, whichever occurred first.

We used receiver operating characteristic (ROC) curves to determine the ability of serum levels of sICAM-1 and sVCAM-1 (Log pg/mL) to predict liver-related events. We also used ROC curves to calculate the optimized cutoff values of sICAM-1 and sVCAM-1, defined as the values with the highest combination of sensitivity and specificity for liver-related events using the Youden J index (J = sensitivity + specificity – 1). We compared the ROC curves following the method of Hanley and McNeil.

The Kaplan-Meier estimator was used to estimate the cumulative probability of events according to the levels of sICAM-1 and sVCAM-1 above or below the optimized cutoff value. We used the Fine and Gray proportional hazards model to test the association between serum levels of sICAM-1 and sVCAM-1 above or below the optimized cutoff value and outcomes; taking into account death as a competitive risk [18]. The covariates for adjustment were age, HIV-transmission category (injection drug use vs. other categories), liver fibrosis (METAVIR F0 to F2 vs. METAVIR F≥3), baseline CD4+ T-cell count, baseline alanine aminotransferase (ALT) serum levels, and SVR following therapy with interferon plus ribavirin (yes vs. no). Statistical analyses were performed using IBM SPSS Statistics for Windows, Version 21.0. Armonk, NY: IBM Corp. The R package was used for plotting the cumulative incidence curves and for competing risks regression analysis [19].

## Results

### Patient Characteristics

The baseline characteristics of the patients are shown in **Table 1**. In brief, 75.3% were male, the median age was 39 years, 32% had prior AIDS-defining conditions, 97.8% were on cART, 11.6% reported a high intake of alcohol, the median baseline CD4+ T-cell count was 480 cells/mm^3^, 81% had an undetectable HIV viral load, 77% were infected by genotypes 1 or 4, and 77% had HCV RNA ≥ 500,000 IU/mL. A total of 32% patients had advanced fibrosis and 11.5% had cirrhosis.

**Table 1:** Characteristics of 182 HIV/HCV coinfected patients who had both a liver biopsy and simultaneous frozen plasma samples taken between 2000 and 2007.

Baseline Characteristic	N = 182
Male sex; n (%)	137 (75.3)
Age–yr.; median (IQR)	39 (37–44)
Prior injection drug use; n (%)	163 (89.6)
Current alcohol intake	21 (11.6)
CDC category C; n (%)	58 (32)
Combination antiretroviral therapy; n (%)	178 (97.8)
Undetectable HIV viral load; n (%)	145 (81)
CD4 + cells baseline–n/mm^3^; median (IQR)	480 (373–648)
HCV genotype; n (%)	
1,4	137/178 (77)
2,3	41/178 (23)
HCV-RNA ≥ 500 000 IU/mL; n (%)	132/171 (77.2)
METAVIR fibrosis stage; n (%)	
F0 or F1	76 (41.8)
F2	48 (26.4)
F3	37 (20.3)
F4	21 (11.5)

All 182 patients were naïve for anti-HCV therapy

### Outcomes

The median follow-up after the liver biopsy was 105 months. During that period, 19 patients had liver-related events, including ascites (n = 10), ascites plus hepatocellular carcinoma (n = 3), hepatocellular carcinoma (n = 2), ascites plus variceal bleeding plus hepatocellular carcinoma (n = 1), ascites plus spontaneous bacterial peritonitis plus variceal bleeding (n = 1), hepatic encephalopathy (n = 1), and hepatic encephalopathy plus hepatocellular carcinoma (n = 1). Liver fibrosis staging at baseline in these 19 patients was as follows: F3, n = 9; F4, n = 7; F2, n = 2; and F0 n = 1.

A total of 16 patients died during follow-up. Causes of death included liver-related death (n = 9), non–liver-related non–AIDS-related death (n = 6), and AIDS-related death (n = 1). Liver fibrosis staging at baseline in these 16 patients was as follows: F4, n = 7; F3, n = 5; and F1-F2, n = 4. Non–liver-related non–AIDS-related death included non–AIDS-related bacterial infections (n = 3), acute myocardial infarction (n = 1), esophageal cancer (n = 1), and suicide (n = 1). The 3 patients who died from non–AIDS-related bacterial infections had F4 at baseline.

During follow-up, 142 patients were treated with pegylated interferon plus ribavirin; of these, 51 achieved SVR. Liver-related events occurred in 12/91 (13%) patients without SVR and in 3/51 (5.9%) patients with SVR (*P* = 0.256). The power to detect a statistically significant difference in liver-related events in patients with and without SVR, for an alpha risk of 5% in a bilateral contrast, was 25%.

### Ability of sCAM-1 and sVCAM-1 to Predict Outcomes

**Fig 1:**
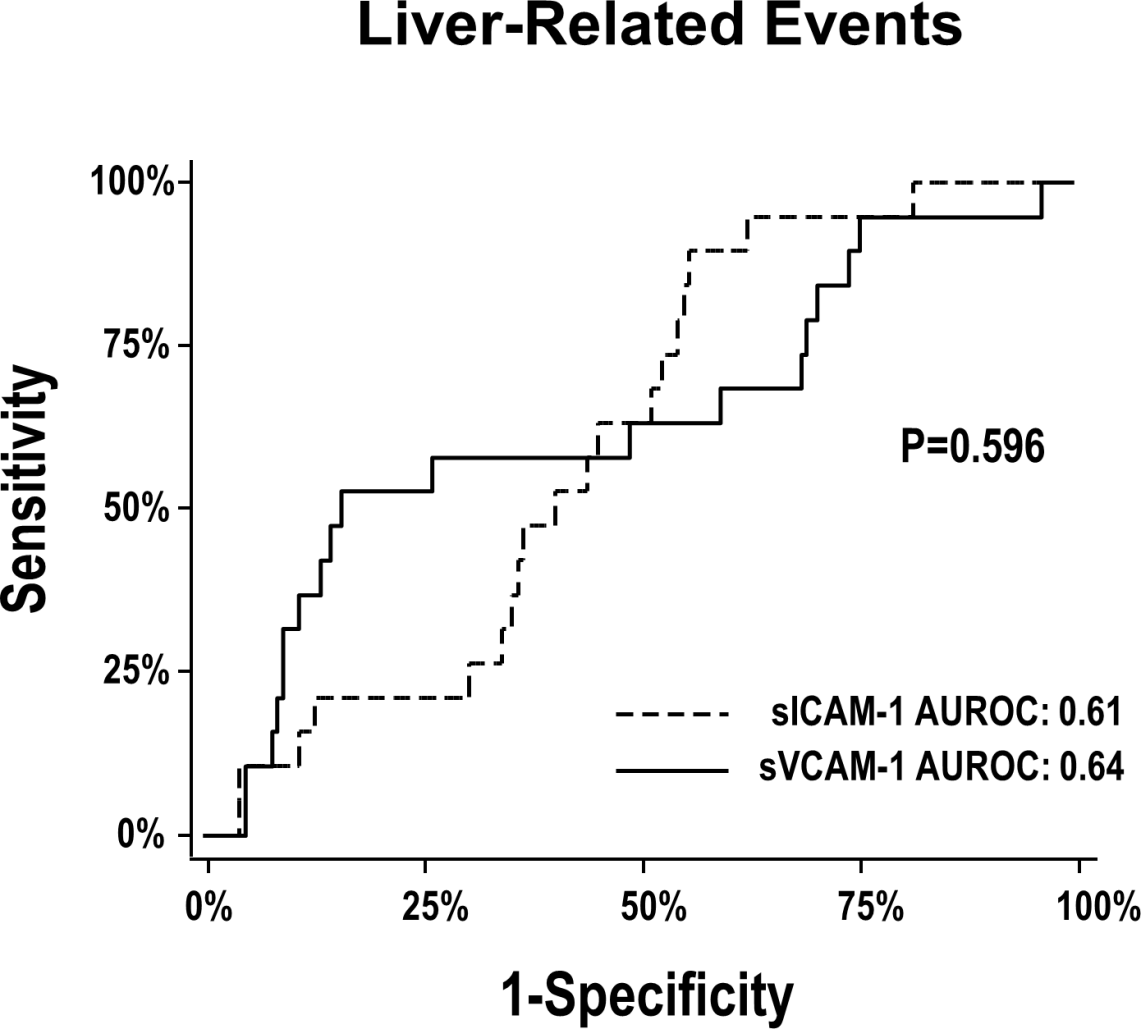
Accuracy of sICAM-1 and sVCAM-1 serum levels for the prediction of liver-related events. The *P* value refers to the comparison of ROC curves according to the method of Hanley and McNeil. Abbreviations: AUROC, area under the receiver operating characteristic curves.

**Fig 2:**
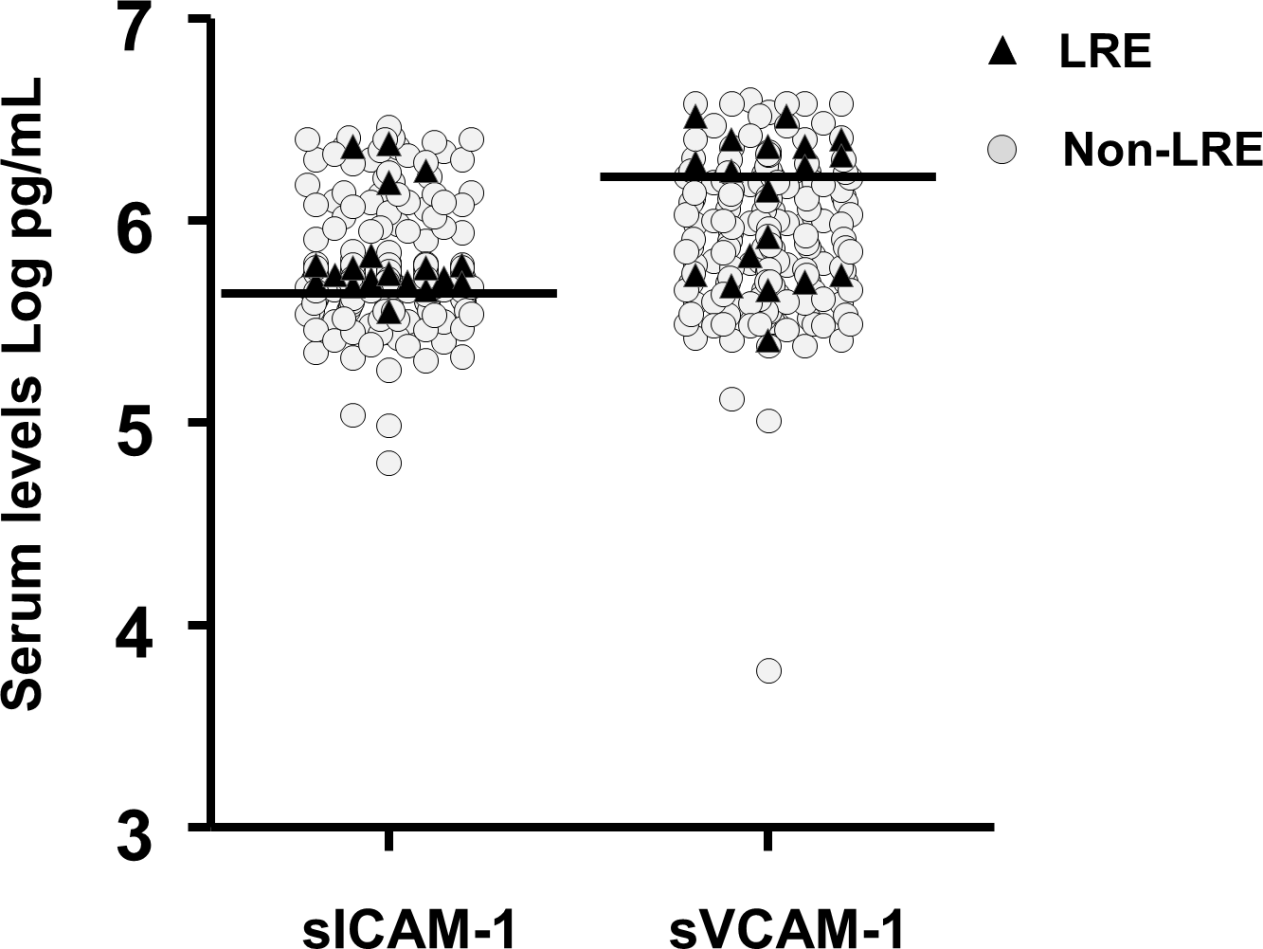
Scatter plot showing the distribution of baseline serum concentrations (Log pg/mL) of sICAM-1 and sVCAM-1 in 182 HIV/HCV coinfected patients included in the study. Each symbol represents a single patient. Triangles represent patients who developed liver-related events during follow-up; circles represent patients who remained free from liver-related events during follow-up. Horizontal bars represent the optimized cutoff values for prediction of liver-related events of sICAM-1 (5.68 Log pg/mL) and sVCAM-1 (6.25 Log pg/mL). Abbreviations: LRE, liver-related events.

The areas under the ROC (AUROC) of serum levels of sICAM-1 and sVCAM-1 to predict liver-related events were 0.61 (95% confidence interval [CI]: 0.50–0.71) and 0.64 (95% CI: 0.49–0.78), respectively (**Fig 1)**; the AUROC of METAVIR fibrosis score to predict liver-related events was 0.81 (95% CI: 0.71–0.91). According to the results of the ROC curves, the optimized cutoff values for prediction of liver-related events were 5.68 Log pg/mL for sICAM-1 and 6.25 Log pg/mL for sVCAM-1, respectively. Distribution of values of sICAM-1 and sVCAM-1 in relation to optimized cutoff values are shown in **Fig 2**.

**Fig 3:**
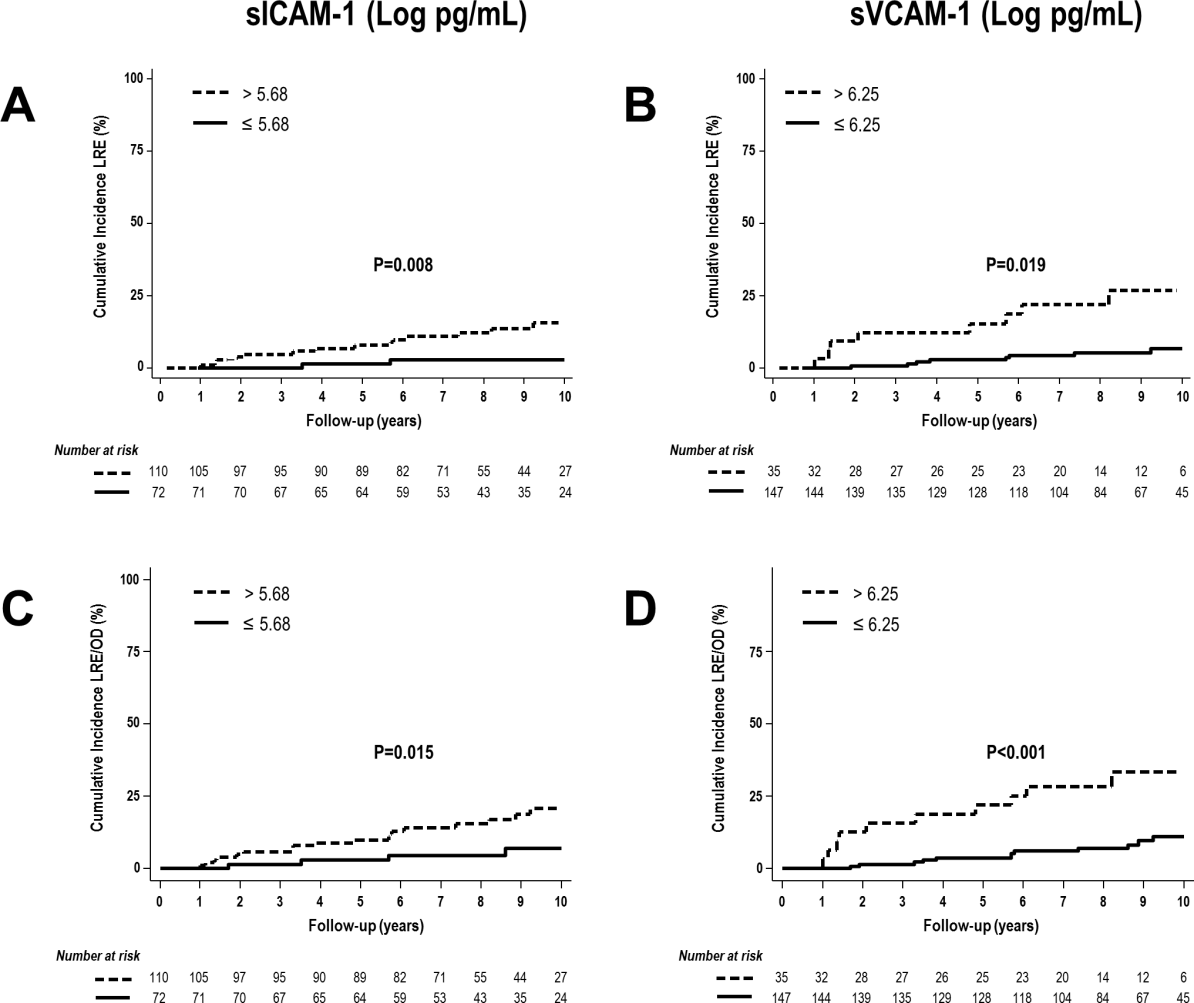
Kaplan-Meier plots showing cumulative incidence of 1) Liver-related events taking into account death as a competitive risk according to sICAM-1 serum levels higher than or lower than or equal to the optimized cutoff values (**A**) and sVCAM-1 serum levels higher than or lower than or equal to the optimized cutoff values (**B**). 2) Liver-related events or death, whichever occurred first, according to sICAM-1 serum levels higher than or lower than or equal to the optimized cutoff values (**C**) and sVCAM-1 serum levels higher than or lower than or equal to the optimized cutoff values (**D**). Abbreviations: LRE, liver-related events; OD, overall death; LRE/OD, liver-related events or death, whichever occurred first.

Kaplan-Meier estimates showed that the probability of liver-related events, and the composite endpoint of death or liver-related events were significantly higher in patients with serum levels of sICAM-1 and sVCAM-1 higher than the optimized cutoff values in comparison with patients with serum levels of sICAM-1 and sVCAM-1 equal to or lower than the optimized cutoff values (**Fig 3**).

Fine and Gray proportional hazard models showed that serum levels of sVCAM-1 above the optimized cutoff value were associated with a significant increase in the adjusted subhazard ratio of liver-related events; something that was close to reaching statistical significance for serum levels of sICAM-1 above the optimized cutoff value (**Table 2**). In addition, serum levels of sICAM-1 or sVCAM-1 above the optimized cutoff values were associated with a significant increase in the adjusted subhazard ratio of the composite endpoint of liver-related events or death, whichever occurred first (**Table 2**).

**Table 2:** Hazard of events for sICAM-1 and sVCAM-1 serum levels higher the optimized cutoff values*.

	Liver-related events		Overall death		Overall death/ Liver-related events	
sICAM-1	aSHR (95% CI)*	P	aSHR (95% CI)*	P	aSHR (95% CI)*	P
sICAM-1 levels Log pg/mL > 5.68 vs. ≤ 5.68	9.92 (0.84;116.84)	0.068	3.26 (0.71;14.85)	0.126	3.98 (1.14;13.89)	0.030
METAVIR F≥3 yes vs. no	23.31 (4.23;202.97)	0.001	11.65 (2.88;47.09)	0.001	11.44 (3.73;35.06)	<0.001
Sustained viral response yes vs. no	0.26 (0.06;1.12)	0.071	0.54 (0.10;2.90)	0.467	0.52 (0.15;1.74)	0.289
Prior or current injection drug use yes vs. no	0.15 (0.02;0.91)	0.040	0.85 (0.09;7.45)	0.886	0.34 (0.07;1.65)	0.185
Age, per year	0.99 (0.91;1.08)	0.946	0.99 (0.89;1.11)	0.951	0.98 (0.90;1.07)	0.787
Baseline CD4+ counts, per 100 cells/mm3	1.12 (0.98;1.00)	0.263	1.06 (0.91;1.23)	0.432	1.04 (0.90;1.20)	0.513
Baseline ALT per each IU/ml	0.99 (0.98;1.00)	0.263	1.00 (0.99;1.00)	0.311	1.00 (0.99;1.00)	0.540
**sVCAM-1**						
sVCAM-1 levels Log pg/mL > 5.68 vs. ≤ 5.68	3.76 (1.16;12.15)	0.027	2.53 (0.78;8.12)	0.118	2.81 (1.10;7.19)	0.030
METAVIR F≥3 yes vs. no	20.60 (3.36;126.02)	0.001	8.29 (2.00;34.36)	0.004	8.08 (2.66;24.53)	<0.001
Sustained viral response yes vs. no	0.30 (0.06;1.49)	0.143	0.56 (0.10;2.92)	0.496	0.56 (0.17;1.86)	0.347
Prior or current injection drug use yes vs. no	0.30 (0.06;1.46)	0.140	1.17 (0.12;10.78)	0.884	0.48 (0.10;2.20)	0.346
Age, per year	1.04 (0.95;1.13)	0.362	1.00 (0.89;1.13)	0.898	1.00 (0.91;1.10)	0.895
Baseline CD4+ counts, per 100 cells/mm3	1.21 (1.05;1.40)	0.010	1.08 (0.91;1.28)	0.221	1.08 (0.92;1.27)	0.313
Baseline ALT per each IU/ml	0.99 (0.98;1.00)	0.270	1.00 (0.99;1.01)	0.221	1.00 (0.99;1.00)	0.405

*Multivariate Fine and Gray proportional hazards model taking into account death as a competitive risk.

aSHR: Adjusted subhazard ratio

## Discussion

Our cohort comprised 182 HIV/HCV-coinfected patients with a baseline liver biopsy and simultaneous frozen plasma samples who were followed for a median of approximately 9 years. We found that high serum levels of sVCAM-1 at baseline were associated with an increased hazard of liver-related events, taking into account death as a competitive risk, and after adjusting for key clinical variables including liver fibrosis and SVR following interferon plus ribavirin therapy. We also found that serum levels of sICAM-1 or sVCAM-1 above the optimized cutoff values were associated with a significant increase in the hazard of the composite endpoint of liver-related events or death, whichever occurred first.

Regardless of etiology, the course of cirrhosis is complicated by systemic inflammation and immunodeficiency, both of which have a critical pathogenic role in liver inflammatory damage, hemodynamic derangements, and clinical manifestations of end-stage liver disease [7, 20]. In addition, serum levels of sICAM-1 and sVCAM-1 are significantly higher in more advanced phases of cirrhosis and have been associated with increased frequency of mortality and liver transplantation in this population group [21, 22]. In one of these works, a sICAM cutoff value of 5.87 Log pg/mL was identified as a good marker for prediction of overall mortality [21]; a value that was close to the optimized cutoff for sICAM of 5.68 Log pg/mL for prediction of the composite endpoint of death or liver-related events identified in our study.

The clinical profiles of the patients in our cohort differ substantially from those included in the studies mentioned above: a small percentage had liver cirrhosis at baseline, although none had decompensated liver disease. Notwithstanding, serum levels of soluble cellular adhesion molecules were found to be predictors of the composite endpoint of liver-related events or death, whichever occurred first; and serum levels of sVCAM-1, were independently associated with an increased hazard of liver-related events taking into account death as a competitive risk.

To better understand these findings, it must be remembered that adhesion molecules play a key role in the recruitment of leukocytes in acute and chronic liver inflammation [23, 24] and that higher serum levels of sICAM-1 and sVCAM-1 have been found in HIV/HCV-coinfected patients with advanced fibrosis and moderate to severe disease activity [14]. In addition, increased baseline inflammatory activity has been associated with a higher frequency of fibrosis progression in serial biopsy studies performed in HIV/HCV-coinfected patients [25]. Our findings are also supported by recent studies that have shown the important role of sVCAM-1 in the pathophysiology of liver disease. Afford et al, carried out ex-vivo studies with T lymphocytes and primary cholangiocytes derived from liver tissue from patients undergoing liver transplantation, and found that VCAM-1 prevented apoptosis of liver-infiltrating T lymphocytes promoting their persistence in the setting of liver inflammation [26]. These findings were supported by the same investigators in a murine model of hepatobiliary inflammation where inhibition of VCAM-1 decreased liver inflammation by reducing lymphocyte recruitment and increasing CD8 and T helper 17 CD4 T-cell survival [26]. Also relevant are the findings of a nested cohort study in the setting of a randomized clinical trial to assess the development of gastroesophageal varices in patients with compensated cirrhosis, in which some inflammatory markers, including sVCAM-1, were significantly correlated with hepatic venous pressure gradient, the best prognostic indicator for long-term survival in cirrhosis [27].

The main limitation of our study is that follow-up information was collected retrospectively. However, we believe that the characteristics of the study make it unlikely that the results differ considerably from those that would have been obtained in an entirely prospective study. This is because follow-up of the patients was carried out in our institution by the same physicians throughout the course of the disease, with standard clinical and laboratory parameters assessed every 3 to 6 months, as is common practice with HIV-infected patients in Spain [28]. In addition, the management and prevention of complications of cirrhosis was conducted following protocols established by the hepatology unit at our institution, based on current clinical practice guidelines.

Our study is also limited by the lack of information about serum levels of sICAM-1 and sVCAM-1 during follow-up and the extent to which SVR could have modified the baseline values of these molecules, as shown previously by our group in a smaller sample [14]. Another limitation is that we did not take cardiovascular risk factors into account, because increased serum levels of sICAM-1 and sVCAM-1 have been independently associated with overall death and death from cardiovascular causes in other population groups, such as those with coronary artery disease and end-stage renal disease [29, 30]. However, only one of our patients died of cardiovascular disease, and nine out of sixteen died of liver disease. In addition, liver disease could have contributed to three additional deaths caused by non–AIDS-related bacterial infections that were, by definition, categorized as non–liver-related non–AIDS-related death but occurred in patients with baseline cirrhosis, which increases susceptibility to bacterial infections [31].

## Conclusions

In conclusion, we found that high serum levels of cellular adhesion molecules were associated with death or liver-related events in HIV/HCV-coinfected patients independently of liver fibrosis stage and SVR. High serum levels of sVCAM-1, in particular, were associated with an increased hazard of liver-related events taking into account death as a competitive risk. These findings lend further support to the potential role of inflammatory biomarkers, including cellular adhesion molecules, and especially sVCAM-1, in assessment of the risk of liver disease progression and mortality in patients with chronic hepatitis C. Furthermore, our results support the notion that despite the major advances in direct-acting antivirals against HCV, therapies targeting necroinflammatory damage and fibrogenesis may have a role in the management chronic hepatitis C.
